# Common and contrasting themes in host cell-targeted effectors from bacterial, fungal, oomycete and nematode plant symbionts described using the Gene Ontology

**DOI:** 10.1186/1471-2180-9-S1-S3

**Published:** 2009-02-19

**Authors:** Trudy Torto-Alalibo, Candace W Collmer, Magdalen Lindeberg, David Bird, Alan Collmer, Brett M Tyler

**Affiliations:** 1 Virginia Bioinformatics Institute, Virginia Polytechnic Institute and State University, Blacksburg, VA 24061, USA; 2 Department of Biological and Chemical Sciences, Wells College, Aurora, NY 13026, USA; 3 Department of Plant Pathology and Plant-Microbe Biology, Cornell University, Ithaca, NY 14853, USA; 4 Center for the Biology of Nematode Parasitism, North Carolina State University, Raleigh, NC 27695, USA

## Abstract

A wide diversity of plant-associated symbionts, including microbes, produce proteins that can enter host cells, or are injected into host cells in order to modify the physiology of the host to promote colonization. These molecules, termed effectors, commonly target the host defense signaling pathways in order to suppress the defense response. Others target the gene expression machinery or trigger specific modifications to host morphology or physiology that promote the nutrition and proliferation of the symbiont. When recognized by the host's surveillance machinery, which includes cognate resistance (R) gene products, defense responses are engaged to restrict pathogen proliferation. Effectors from diverse symbionts may be delivered into plant cells via varied mechanisms, including whole organism cellular entry (viruses, some bacteria and fungi), type III and IV secretion (in bacteria), physical injection (nematodes and insects) and protein translocation signal sequences (oomycetes and fungi). This mini-review will summarize both similarities and differences in effectors and effector delivery systems found in diverse plant-associated symbionts as well as how these are described with Plant-Associated Microbe Gene Ontology (PAMGO) terms.

## Effectors from diverse plant-associated symbionts

Diverse organisms live in intimate association with plants, with the outcome of these associations dependent upon a complex interplay of gene products. Among the most significant of these are the effector proteins, defined as molecules deployed by symbiotic organisms that manipulate host cell structure and function, and thereby facilitate symbiont success [1]. In some cases, through the action of the host surveillance machinery, effectors trigger defense responses; in that context, effectors have historically been called avirulence factors or elicitors. In fact, the detection of effectors by the products of host resistance (R) genes has been central to the identification of effectors in diverse symbionts (reviewed in [2,3]). This particular review will focus on properties of effector proteins that enter the host cytoplasm and the role that Gene Ontology (GO) can play in highlighting similarities and differences exhibited by effectors deployed by plant pathogens from diverse biological kingdoms.

It is important to note that while this review focuses on organisms living in a pathogenic relationship with the host plant, there are many associations that cannot readily be identified as beneficial or antagonistic to the host because the outcome depends on the context in which it occurs. For example, while some rhizobacteria are pathogenic, their colonization of plant roots can also play a beneficial role by priming plant defense responses, thus making the plant more resistant to infection by unrelated pathogens. As a result, the term "symbiont" is used by the GO and in this review to describe organisms living in intimate association with a larger host organism, irrespective of whether the association may be beneficial or antagonistic. The Gene Ontology Consortium (GOC) strongly discourages the use of the word symbiosis as a synonym for mutualism. Symbionts may be microbes (for example bacteria, fungi or oomycetes) or they may be more complex multicellular organisms such as nematodes, insects or parasitic plants.

Many gram-negative bacterial symbionts, including mutualists of the genus *Rhizobium *and pseudomonad and xanthomonad pathogens, utilize a molecular needle created by the type III or type IV secretion systems to deliver effectors into the host cell (reviewed in [4-6]). Most progress in effector characterization has been made with the gram-negative bacterial pathogens. The sequencing of gram-negative bacterial genomes has further advanced the discovery of effectors by enabling bioinformatic identification of new candidate effectors [7,8]. Bioinformatic analysis of genome sequences has also greatly advanced the identification of the effectors produced by obligate symbionts such as gram-positive phytoplasmas [9].

Oomycete and fungal pathogens represent different kingdoms of life but share similar strategies in colonizing their hosts, presumably as a result of convergent evolution [10]. Biochemical and genetic approaches have identified effectors from both taxa (reviewed in [1,11-15]). Given the predicted role of the haustorium, a differentiated feeding structure produced by both fungi and oomycetes [16,17], as a site of effector release, identification of haustorially expressed secreted proteins (HESPs) has proven to be a valuable source of candidate effectors [18,19]. Genome sequences of fungal and oomycete pathogens have dramatically accelerated the discovery of effectors via bioinformatic analyses of predicted secretomes [20-25]. In particular, the discovery of the protein transduction motif RXLR-dEER [25-27] enabled the identification of hundreds of effector candidates in oomycete genomes [21,24,28].

Nematodes comprise a large phylum of animals that include free-living species as well as plant and animal parasites. Most plant pathogenic nematodes are obligate parasites and obtain nutrients from the cytoplasm of living root cells. The sedentary endoparasites of the family Heteroderidae, which include members of the genera *Heterodera *(cyst nematode) and *Meloidogyne *(root knot nematode) cause the most economic damage worldwide. Infection by these pathogens is characterized by the release of esophageal gland secretions via a hollow protrusible stylet [29]. During nematode migration, cell wall degrading enzymes [30,31] are released into the apoplast in amounts sufficiently copious to be visible under the light microscope [32]. Upon becoming sedentary, other proteins, including plant peptide hormone mimics [33], are delivered to those cells destined to become the feeding sites. This occurs via fusion of neighboring cells (for cyst nematodes) or via repeated nuclear division (in the case of root knot nematodes). It is presumed that nematode proteins, sometimes called parasitism proteins, are introduced both onto the membrane surface of the targeted plant cells, and also directly into the cytoplasm.

Effectors from diverse microbes have little in common at the sequence level, but as a result of convergent evolution, may implement common strategies in defeating host defenses. Therefore, in order to carry out functional comparisons of diverse effectors, an approach is required that does not depend on sequence similarities. The GO provides such an approach. Established in 1998, the GO provides a uniform language to describe attributes of gene products from all organisms in the context of their molecular function, biological process, and cellular location [34,35]. The Plant-Associated Microbe Gene Ontology (PAMGO) consortium [36] was established in 2004 to develop GO terms to describe common biological processes utilized by symbionts (particularly microbes) in their interactions with hosts. The current count of terms created via the PAMGO effort is over 700. To create well-annotated reference genomes that provide high quality examples of the usage of the new terms, the consortium has been using the terms to annotate the genomes of the bacteria *Pseudomonas syringae *pv *tomato *DC3000, *Dickeya dadantii (Erwinia chrysanthemii) *3937, and *Agrobacterium tumefaciens; *the fungus *Magnaporthe oryzae (M. grisea); *and the oomycete *Phytophthora sojae*.

This review focuses on the effectors and effector delivery systems of diverse plant-associated microbes and nematodes with an emphasis on pathogens. Similarities and differences in pathogen-host associations with respect to the role of effectors are described in the context of GO terms that best describe them. This is by no means a comprehensive coverage of the subject due to space limitations, but rather is intended to illustrate the value of using the GO for comparative genome analyses of diverse symbionts.

## How are effectors introduced into host cells?

**Figure 1:**
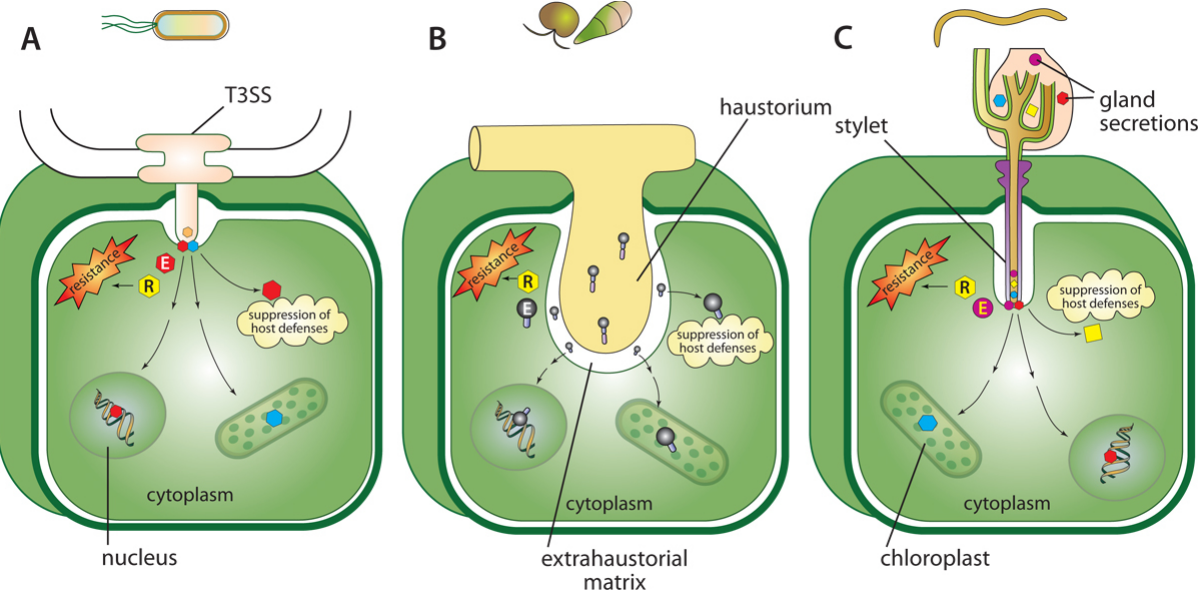
**Effector delivery structures of Gram-negative bacterium, oomycete, fungus, and nematode in plant cell**. **(A) **Type III secretion system in Gram-negative bacterium injects effectors into the host cell. **(B) **The haustorium in biotrophic and hemibiotrophic filamentous pathogens is believed to be the site of effector release into the host cell. **(C) **Gland secretions, which include effectors, are injected into the plant cell via the stylet of the nematode. Effectors (E) thus delivered, can either suppress host defenses and/or trigger host cell defenses, which include programmed cell death (PCD) upon recognition by resistance (R) proteins. Recognition of effectors by R proteins may occur directly (observed with some fungal effectors) or indirectly as a result of interaction of the effectors with other host protein(s) (observed with a number of bacterial effectors). Potential subcellular locations of effectors such as the nucleus and chloroplasts are also shown.

Critical to effector function is their successful delivery to their site of action in the host cell. For the pathogens discussed here, this process involves passage across the plant cell wall and the plasma membrane. The injectisomes of bacterial type III and type IV secretion systems (T3SS and T4SS) respectively; (reviewed in [6,37-39]) are analogous to the stylets of plant parasitic nematodes. Also known as the Hrp pilus, the T3SS injectisome spans both the bacterial envelope and the plant cell wall, forming a channel between the bacterial cytoplasm and the host plasma cell membrane. Secreted proteins delivered by the injectisome then form a pore through the membrane that enables translocation of effector proteins into the host cell (Figure 1a) [5]. The stylet in nematodes executes an analogous function, in that it mechanically pierces the host cell wall but not the membrane and injects gland secretions, including effectors, into the host cell cytoplasm via an orifice at the tip of the stylet (Figure 1c) [31,40].

In the case of many biotrophic and hemibiotrophic fungi and oomycetes, penetration of the host cell wall is accomplished via a hypha that differentiates into a specialized feeding structure called a haustorium (in the case of pathogenic fungi and oomycetes) or an arbuscle (in the case of mutualistic arbuscular mycorrhizal fungi). The haustorium becomes surrounded by a specialized interface consisting of the plasma membranes of the pathogen and host separated by a modified pathogen cell wall (Figure 1b) [41,42]. The haustorial interface is speculated to be the site of nutrient acquisition as well as the site of effector release from the pathogen into the plant tissue [16], though the mechanism of subsequent effector transfer across the plasma membrane remains uncharacterized.

The GO provides terms to describe gene products involved in the formation of these effector delivery structures, the gene products aiding in the delivery of effectors, and the gene products (effectors) that are delivered through these structures. The PAMGO Consortium has contributed many of these terms. [10,43,44]. We use the T3SS as an illustration. Gene products encoding the structural components of the T3SS injectisome may be annotated with the cellular component term "GO:0030257 type III protein secretion system complex". Furthermore, gene products that are involved in the secretion of effectors into the host cell, including helper proteins such as chaperones and harpins may be annotated with the process term, "GO:0030254 protein secretion by the type III secretion system". The term "GO:0052049 interaction with host via protein secreted by type III secretion system" may be used to annotate all gene products that are secreted via the T3SS and that interact with the host. These will include harpins and effectors delivered via the T3SS. Additionally the effectors may be annotated with the GO cellular component term "GO:0043657 host cell" to indicate the site of interaction with the host. A direct parent term of "GO:0052049 interaction with host via protein secreted by type III secretion system" is "GO:0052048 interaction with host via secreted substance" which is in turn a child term of "GO:0051701 interaction with host". As basis for comparison, a new sibling term to GO:0052049, "interaction with host via protein secreted by the stylet" has been created for annotation of nematode effector proteins.

The exact mechanism by which oomycete and fungal effectors enter plant cells is not clear, though the haustorial interface is speculated to be the site of entry. Recent studies of two oomycete effectors, Avr1b from *P. sojae *and Avr3a from *P. infestans *have identified the motif RXLR-dEER, present in the N-terminus of both proteins, as being necessary and sufficient to deliver proteins into the plant cell [25,26]. Although RXLR-dEER-bearing proteins could cross the plasma cell membrane autonomously, some evidence suggests that entry may be more efficient at the haustorium, where the plant cell wall was penetrated [26], emphasizing the analogy of the haustorial hypha with the T3SS injectisome and the nematode stylet.

Subsequent to characterization of Avr1b and Avr3a, a super-family of 385 RXLR dEER proteins in the *P. sojae *genome and 370 in the *P. ramorum *genome was identified using bioinformatic approaches such as recursive BLAST and HMM searches [21]. The existence of this predictive motif among oomycete effectors with varying levels of experimental characterization can be used to highlight the importance of evidence codes in GO annotation. Given the experimental evidence, the *Phytophthora *Avr1b and Avr3a gene products can be annotated with "GO:0052048 interaction with host via secreted substance" with an experimental evidence code. Once a specific structure or mechanism is identified through which the effectors are delivered, a more specific child term will be created and applied. Given the presence of the RXLR-dEER motif in the bioinformatically characterized proteins, it is appropriate to infer that like Avr1b, these proteins are also targeted to the host cell and can be annotated to "GO:0052048 interaction with host via secreted substance". However, in these cases the annotation would be accompanied by the evidence code "Inferred from Sequence Model" (ISM) with the Avr1b protein accession documented as the experimentally characterized effector.

## Where do they lay camp when in the host?

Prokaryote and eukaryotic pathogens alike secrete effector proteins into the host apoplast as well as into host cells where they may localize to the cytoplasm and subcellular compartments, including the mitochondrion, nucleus and the chloroplast. Specific terms were developed by the PAMGO consortium under the cellular component ontology to describe gene products from one organism (symbiont) that act in the extracellular and cellular regions of another organism (host) cell. These terms are different from terms developed to describe gene products from an organism acting in cellular locations within the same organism. Gene products from one organism acting in regions of another organism are described with "GO:0043657 host cell" and its child terms. The term host cell has a "part-of" relationship with the parent term "GO:0018995 host" which in turn is a child term of "GO:0043245 extraorganismal space". In contrast, gene products from one organism acting in regions of that same organism are captured under "GO:0044464 cell part" and its child terms. "Cell part" has a part of relationship with "GO:0005623 cell" which is a direct child of the root "GO:0005575 cellular component". We illustrate the use of these terms with gene products from diverse organisms. For example, "GO:0043655 extracellular space of host" can be used to describe microbial gene products secreted into the apoplast of plant cells while "GO:0005615 extracellular space" is used to describe microbial gene products shown to be located outside of the microbe's plasma membrane. Apoplastic effectors are secreted into the plant extracellular space where they interact with extracellular targets and surface proteins. For example, plant cell wall-degrading enzymes secreted by bacterial, fungal, oomycete and nematode pathogens could be annotated with "GO:0043245 extraorganismal space".

Many effectors from bacterial, fungal, oomycete and nematode pathogens can enter the cytoplasm of host cells, and could be annotated with the term "GO:0030430 host cell cytoplasm" unless a more specific location was identified. In some cases, the evidence for host cytoplasmic location is indirect, for example, some effector proteins are recognized by intracellular plant disease resistance gene products [45]. In other cases the evidence for cytoplasmic localization is directly supported by experimental evidence showing physical interactions between effectors and resistance gene products or other proteins in the plant cytoplasm. Examples include the *Magnaporthe oryzae *effector AvrPita which interacts with the rice resistance gene product Pita [46]. In other cases, effector proteins have been identified in the plant cell cytoplasm cytologically: by antibody staining or via a fluorescent tag. These include, for example, the bacteria effectors, HopAB2 [47] and HopU1 [48]; and the oomycete effectors Avr1b [26] and Avr3a [49].

Some intracellular effectors have also been located in specific host organelles, including the nucleus and chloroplast, and thus can be annotated with "GO:0042025 host cell nucleus" or "GO:0033652 host cell chloroplast" respectively. Examples of nucleus-located effectors include AvrBs3 and other members of the AvrBs3 family from *Xanthomonas *bacteria [50], the rust transferred protein 1 (Uf-RTP1p) from the fungus *Uromyces fabae *[51], four putative effectors from the oomycete *Phytophthora infestans *(Nuk6, Nuk7, Nuk10, Nuk12) [52], and two nematode parasitism proteins [53]. An example of a chloroplast-located effector is HopI1 [54].

## What effectors do in the host

Plants have evolved mechanisms to passively withstand or actively resist invading microbes by deploying defense responses. Defense responses may be triggered by plant recognition of commonly occurring pathogen molecules called pathogen-associated molecular patterns (PAMPS) such as bacterial flagellin (PAMP triggered immunity; PTI) or by direct or indirect recognition of pathogen effectors (effector triggered immunity; ETI) (reviewed in [55,56]).

An important process associated with defense against biotrophic and hemibiotrophic pathogens is programmed cell death (PCD). Many pathogen effectors have been demonstrated to suppress PCD. Among these are HopAB2 (AvrPtoB) from *P. syringae *[57] and oomycete effectors such as *Phytophthora sojae *Avr1b [58], which have been shown to inhibit defense-like PCD triggered in plants by other effectors or by the pro-apoptotic mammalian BAX protein. Similarly, the *P. infestans *effector AVR3a^KI ^can suppress PCD triggered by the PAMP, INF1 in *Nicotiana benthamiana *[59]. These effectors can be annotated with "GO:0034054 negative regulation by symbiont of host defense-related programmed cell death". In contrast to biotrophs and hemibiotrophs, necrotrophs induce PCD in order to colonize their host [60]. For example, the Nep1-like protein NPP_Ps _(previously called PsojNIP) from the hemibiotrophic oomycete pathogen *P. sojae *causes necrosis in soybean. Its expression during the transition from biotrophy to necrotrophy [61] suggests its effector role is to manipulate PCD to the advantage of the pathogen. This role can be described jointly with the two GO terms "GO:0052042 positive regulation by symbiont of host programmed cell death" and "GO:0009405 pathogenesis".

The specific processes that contribute to ETI and PTI are complex and many of their details remain a mystery. However, ongoing characterization of individual effectors has revealed new insights into the various defense mechanisms deployed by the host and subject to interference by the symbiont. One method of defense suppression involves inactivation, modification, or suppression of host defense proteins. For example, XopD and AvrXv4 from *Xanthomonas campestris *are cysteine proteases that have been predicted to remove SUMO (small ubiquitin-like modifier) modifications from components of the defense pathways (reviewed in [62]). The *P. syringae *effectors AvrRpt2 and HopAR1 (AvrPphB) also function as cysteine proteases [63,64] while the fungal effector AvrPita from *Magnaporthe oryzae *is a zinc metalloprotease [65]. These effectors can be annotated with the term "GO:0052014 catabolism by symbiont of host protein".

Inhibition of host hydrolytic enzymes is another mechanism by which effectors interfere with the functions of host defense proteins. For example, the extracellular fungal effectors Avr2 and Avr4 from *Cladosporium fulvum *can inhibit the tomato extracellular protease, Rcr3 [66], and host chitinases [67] respectively. In oomycetes, the glucanase inhibitor protein (GIP1) secreted by *P. sojae *inhibits endoglucanse ability of the plant host [68] and apoplastic effectors EPI1 and EPI10 from *P. infestans *inhibit the P69B subtilase of tomato [69,70]. These host hydrolase inhibitors can be described with "GO:0052053 negative regulation by symbiont of host enzyme activity".

Hallmarks of PTI include not only deployment of defense proteins but also deposition of callose in the host cell wall. Several bacterial and oomycete effectors have been shown to suppress callose deposition *in planta*, including AvrE [71], AvrPto [72], HopM1 [71] from *Pseudomonas syringae*, DspA/E [73] from *Erwinia amylovora *and ATR1 from the oomycete *Hyaloperonospora arabidopsidis *[74]. The appropriate GO term to describe this virulence function is "GO:0052087 negative regulation by symbiont of defense-related host callose deposition".

The various defense responses involved in a successful immune response are dependent on an array of signaling pathways that link pathogen detection to host response. These defense signals include the hormone ethylene, jasmonic acid, and salicylic acid with each representing a target for interference by symbiont effectors. For example, bacterial effectors AvrB and AvRpt2 [75] have been shown to trigger the expression of the ethylene-responsive transcription factor (RAP2.6) in *Arabidopsis *via jasmonic acid signaling thereby repressing salicylic acid (SA) mediated PAMP-triggered defense responses against biotrophic pathogens. The phytotoxin, coronatine from *P. syringae *mimics jasmonic acid also leading to repression of SA signaling [76]. In other cases, hormone signaling is disrupted for the purpose of modifying host morphology. The *Meloidogyne javanica *chorismate mutase 1 (MjCM-1) [77], is secreted into plant cells where it reduces the synthesis of auxins, flavanoids, SA and phytoalexins. A general term for describing effectors that modulate hormone signaling is "GO:0052027 modulation by symbiont of host signal transduction pathway", while a more specific term to describe interference with the host salicylic pathway is "GO:0052003 negative regulation by symbiont of defense-related host salicylic acid-mediated signal transduction pathway ".

Though a direct role in virulence beyond defense suppression remains elusive for most microbial effectors, esophageal gland secretions translocated into host cells via the nematode stylet play major roles in modification of host cells for feeding and pathogenesis [78]. In particular, the *Heterodera glycines *effector HG-SYV46 acts as a functional analog of the plant cellular proliferation regulators that include CLAVATA3 [33]. Effectors such as HG-SYV46 with a demonstrated role in inducing the modification of these plant cells can be annotated with the term "GO:0044005 induction by symbiont in host of tumor, nodule, or growth" which is a child of "GO:0044003 modification by symbiont of host morphology or physiology". Another annotation could be made using "GO:0052096 formation by symbiont of syncytium involving giant cell for nutrient acquisition from host", a child term of "GO:0052093 formation of specialized structure for nutrient acquisition from host".

Though effectors have proven highly effective in suppression of plant defense, the fact remains that in the ongoing arms race between host and symbiont, hosts have evolved successful means of detecting many of the known effectors, most notably through deployment of resistance (R) proteins. Effectors recognized directly or indirectly by R proteins have been termed avirulence proteins and include (among many others) the bacterial effectors AvrPto, AvrRpt2, and AvrRpm1 (reviewed in [79]), the oomycete effectors Avr1b, Avr4, ATR13 and ATR1^Nd ^(reviewed in [15]), the fungal effectors Avr-Pita, AvrL567, AvrM, AvrP123 and AvrP4 (reviewed in [12,13]) and the nematode effectors map1 [80] and Cg-1 [81]. The induction of defense responses by these effectors can be annotated with "GO:0052509 positive regulation by symbiont of host defense response" or if a resistance gene has been identified, "GO:0052527 positive regulation by symbiont of host resistance gene-dependent defense response". If host defense-related programmed cell death is involved, annotation can be made to "GO:0034055 positive regulation by symbiont of host defense-related programmed cell death". Note that these terms differ from "GO:0052042 positive regulation by symbiont of host programmed cell death" which is used to annotate toxins produced by some necrotrophs. It could be argued that positive regulation by the symbiont of the host defense response is deleterious to the symbiont, and hence is not a natural symbiont process. However, what is deleterious to the symbiont can be highly dependent on the context (just as "pathogenicity" is highly context-dependent) with regard to the bio/necro-trophic nature of the interaction. Thus the GO does not attempt to describe the outcome of symbiont processes. An ongoing initiative in the GO in the context of host-symbiont interactions is to create a mechanism to record information about the actual host protein (e.g., an *R *gene product) that mediates the response to a particular effector. Currently there is no way to record interacting proteins in the GO unless the experiment involves direct physical interactions where the "Inferred from Physical Interaction" (IPI) evidence code (see [82] for more information on GO evidence codes) can be used. However, at the current time all the annotations described above where effectors are secreted and act in the host organism would be accompanied by the taxon ids of both the microbe and the plant host.

Overall, modifications made to the host, either by triggering host defenses and/or suppressing host defenses can be described under the broad term "GO:0044003 modification by symbiont of host morphology or physiology". The child terms under GO:0044003 can be used to describe specific effector modifications in the host.

## Conclusion

The value of GO annotations in efficiently summarizing information about gene products from the literature in a standardized way cannot be over-emphasized. Careful GO annotations enable the systematic synthesis of both accumulating sequences from genome projects and advances in studies on effector biology, which provides a wealth of data that is easily accessible to the scientific community. The GO terms developed by the PAMGO consortium greatly improve the resources for annotation of diverse symbiont genomes, particularly for gene products such as effectors that are directly involved in the interaction with the host. Such annotations can be used to aid interpretation of genome sequence comparisons and of microarray and proteomics data. Increased community involvement in GO annotation of more symbiont genomes, along with the development of additional GO terms, will provide valuable resources for more comprehensive cross-kingdom effector analyses, which ultimately will lead to a better understanding of mechanisms underlying symbiont interactions with hosts.

## Competing interests

The authors declare that they have no competing interests.
